# Life-saving effect of pulmonary surfactant in premature babies

**DOI:** 10.1172/JCI179948

**Published:** 2024-05-01

**Authors:** J. Usha Raj, Richard D. Bland, Jahar Bhattacharya, Marlene Rabinovitch, Michael A. Matthay

**Affiliations:** 1 Department of Pediatrics, University of Illinois at Chicago, Chicago, Illinois, USA.; 2 Department of Pediatrics, Stanford University, Stanford, California, USA.; 3 Department of Medicine, Columbia University, New York, New York, USA.; 4 Cardiovascular Research Institute, Departments of Medicine and Anesthesiology, San Francisco, California, USA.

## Abstract

The discovery and replacement of lung surfactant have helped increase survival rates for neonatal respiratory distress syndrome in extremely premature infants.

In 1924, when the *Journal of Clinical Investigation* was founded, babies born prematurely with immature lungs rarely survived. Most of them died soon after birth, struggling to breathe, and no one knew why. Kurt von Neergaard recommended in a 1929 report that “Surface tension as a force counteracting the first breath of the newly born should be investigated further” (1).

## Surface-active material

Nearly three decades later, two seminal articles were independently published by Richard Pattle and John Clements on the surface tension properties of the lung’s alveolar lining liquid (2,3). These studies related the unique pressure volume characteristics of mature lungs to the surface-active material in the lung. However, it was Mary Ellen Avery, a research fellow at Harvard, who made the connection between lack of this surface-active material and the high surface tension of lung liquid extracts from premature babies who had died. She went to work with John Clements, who was using a modified Wilhelmy surface balance plate to measure the surface tension of liquids. Avery and her research mentor at Harvard, Jere Mead, published their landmark article on the association of high surface tension of lung liquid, lung atelectasis, and hyaline membrane disease, now known as neonatal respiratory distress syndrome (RDS) (4).

John Clements moved to the University of California San Francisco and, with coinvestigators, published the first article on the chemical composition of surfactant in 1961 (5). Over a decade later, in 1973, King et al. (6) identified, for the first time, a surfactant-associated apoprotein (SP-A) that was concentrated 50-fold in the surface active material (6).

## A surge to find a cure

In 1963, the death of President John and Jackie Kennedy’s prematurely born son, Patrick Bouvier Kennedy, from RDS led to a surge in research to find a cure. Soon thereafter, clinical trials were initiated using synthetic surfactant phospholipids, the main component of surfactant, but early trials failed. At the same time, George Brumley and colleagues (7) measured diphosphatidyl choline (DPPC) per milligram of DNA in fetal lamb lungs and observed an inverse correlation among minimum surface tension values at different stages of maturation compared with the same measurements made in lungs of term newborn and adult sheep, showing that term newborns’ lungs had three times greater DPPC levels per milligram of DNA than preterm fetal lungs. Notably, the ratio correlated with lower minimum surface tension measurements in lungs of lambs born at term than in lungs of preterm fetuses.

A very important clinical development in the management of newborns with surfactant deficiency and associated respiratory distress came in the early 1970’s, when Gregory and colleagues used a simple device to transmit positive pressure to the lungs of premature babies, now known as continuous positive airway pressure (CPAP), and reported that maintaining the lungs distended with CPAP improved survival of newborns with RDS (8). Thus, CPAP saved the lives of many babies well before surfactant treatment became available. Clements and his research group’s observation that lung inflation was important for the release of surfactant from alveolar epithelial cells helped explain both the efficacy of constant distending pressure as therapy for RDS and use of sustained inflation during resuscitation of preterm babies in the delivery room (9). His group, which included obstetricians, pediatricians, pulmonologists, and neonatologists, developed a rapid bedside test of amniotic fluid to predict susceptibility to RDS (10). Obstetricians still use this test to help make decisions about the timing of delivery of premature babies, and pediatricians use it to anticipate the needs for postnatal care.

Following the initial identification of a surfactant-associated apoprotein by King et al. (6), a series of major advances came from the laboratory of Jeffery Whitsett’s research group, who identified the presence of important, unique proteins associated with surfactant (11). They found that the main function of SP-B) is to increase the adsorption rate of phospholipids at the air-liquid interface, and, via its interaction with SP-A and calcium, SP-B also helps regulate formation of tubular myelin — the highly organized lattice-like structure of surfactant within the lung’s alveolar lining liquid. SP-B, by stabilizing the alveolar surfactant film, increases surfactant’s resistance to inhibition by plasma proteins contained in pulmonary edema fluid. Rapid dispersion of surfactant on the alveolar liquid lining after its release from alveolar type 2 epithelial cells and recycling of surfactant within the lung are attributed to surfactant proteins B and C. Cloning of the surfactant protein genes further accelerated progress made in understanding their respective functions. A *JCI* review of progress in studies of surfactant and its apoproteins was published in 1990, reporting the specific functional contributions of SP-A, SP-B, and SP-C, and emphasizing that SP-C was only produced by alveolar type 2 cells (12).

Liggins and Howie’s original report on the effect of antenatal glucocorticoid treatment to enhance lung maturity and thereby prevent neonatal RDS (13) was followed by over thirty years of research on use of antenatal glucocorticoids to induce fetal lung maturation, (14) including evidence that glucocorticoids increase mRNA expression of SP-B and SP-C (15). Treatment with antenatal glucocorticoids remains a principal treatment for enhancing lung maturation for preterm infants.

The clinical breakthrough for treatment of premature newborns with RDS came in 1980, when Fujiwara and colleagues reported the beneficial effects of a modified bovine surfactant in ten preterm babies (16). In 1987, Clements and colleagues reported on the beneficial impact on lung function of premature rabbits treated with a synthetic surfactant that they had developed (17). They named it Exosurf, which was composed of a mixture of surfactant lipids (plus hexadecanol and tyloxypol to facilitate adsorption and spreading of the surfactant lipids), without surfactant apoproteins. Soon thereafter, the first clinical trials using either artificial or natural surfactants started. One of us, a neonatologist (JUR), in 1987 truly appreciated the “miracle” of surfactant when her prematurely born son, born with RDS, became cyanotic, requiring assisted ventilation with oxygen. He was treated “compassionately” with surfactant, which was not yet FDA-approved, and rapidly turned pink, able to breathe on his own in room air within 12 hours. The first FDA approval for surfactant treatment was granted in 1989 for the artificial surfactant, Exosurf (18). A year later, the FDA approved natural calf lung–derived surfactant, Survanta (19). A series of randomized clinical trials followed using various surfactants, derived from natural sources, which contained surfactant proteins. A comparison of natural bovine surfactant versus artificial surfactant showed that the natural surfactant had a better clinical effect and less morbidity than did artificial surfactant (20).

Pulmonary edema is a prominent finding in neonatal RDS. Usha Raj made direct micropuncture measurements of alveolar liquid pressure in isolated lungs of preterm rabbits and reported an association of surfactant deficiency and high surface tension that was linked to low interstitial fluid pressure, thereby yielding edema in their immature lungs (21). In a related study of preterm neonatal sheep that were mechanically ventilated for up to eight hours after birth, it was reported that pulmonary edema with increased lung vascular protein permeability was a prominent pathological feature of the RDS that developed in the surfactant-deficient premature lambs (22). A subsequent study by the same investigators showed that surfactant treatment of mechanically ventilated premature lambs prevented both the lung edema and the increased lung vascular protein leak that developed in control ventilated animals that did not receive surfactant (23).

The impressive beneficial effects of surfactant therapy in premature newborns led to clinical trials of surfactant in the Acute Respiratory Distress Syndrome (ARDS), a syndrome of acute respiratory failure, most commonly from pneumonia or sepsis, in which surfactant function is inhibited by the presence of proteins in the exudate within the alveoli (24). Several trials were conducted using exogenous administration of surfactant in ARDS patients. These studies showed a small improvement in oxygenation, but no reduction in mortality (25). Hence, surfactant replacement is not used for treatment of ARDS, largely because the mechanisms of lung injury in ARDS do not depend primarily on surfactant deficiency, but rather on alterations in lung endothelial and alveolar epithelial permeability (26).

## Looking forward and back

Continued research is needed to identify ways to store and administer surfactant to the vast number of premature babies born in underdeveloped countries who do not have access to this life-saving therapy. Ongoing research aimed at developing a surfactant that could be stored at room temperature and that could be aerosolized to treat neonatal RDS without the need for endotracheal intubation or assisted ventilation currently shows considerable promise (27).

**Figure 1:**
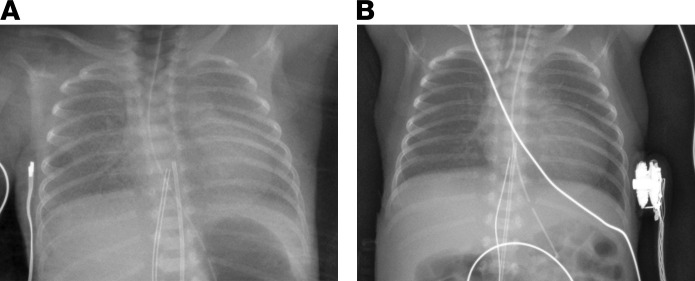
Chest radiographs of an intubated preterm infant with neonatal RDS exemplifies the benefits of surfactant treatment. (**A**) Before treatment, the baby was receiving CPAP with a fractional inspired oxygen concentration (FiO2) of 0.7. The classical radiographic findings showed hypoaerated lungs and air-filled bronchi, indicated by a light lung appearance with a fine granular pattern. (**B**) After intratracheal exogenous surfactant treatment, bilateral lung aeration improved with a reduction in FiO2 to 0.25. These improvements were reflected by darker lung appearance in the radiograph image. We thank Shoshana Newman-Lindsay and Francis Poulain at the University of California at Davis for providing these radiographic images, which were obtained with written informed consent.

It is especially noteworthy that replacement of lung surfactant has helped to increase survival rates for extremely premature infants with neonatal RDS (those weighing under 1,500 grams at birth), from approximately five percent in the 1960s to upwards of 90 percent today (28). As noted by John Clements and Mary Ellen Avery in their 1998 review of lung surfactant and neonatal RDS (29), the National Heart Lung Blood Institute and the National Institutes of Child and Human Development provided crucial support for the research done on surfactant and surfactant therapy, which remains one of the greatest scientific achievements of these institutes (Figure 1).

## Version 1 05/01/2024

Electronic publication

## Version 2 05/01/2024

Corrected typo
